# Advanced polymeric matrix utilizing nanostructured bismuth and tungsten oxides for gamma rays shielding

**DOI:** 10.1016/j.heliyon.2024.e37289

**Published:** 2024-08-31

**Authors:** Hamoud Kassim, Saad Aldawood, Saradh Prasad, Nassar N. Asemi, Aziz A. Aziz, Mohamad S. AlSalhi

**Affiliations:** Department of Physics and Astronomy, College of Science, King Saud University, P. O. Box-2455, Riyadh, 11451, Saudi Arabia

**Keywords:** Gamma radiation protection, Polymer composites, Nanoparticle fillers, Attenuation coefficients

## Abstract

In this study, the shielding properties of novel polymer composites, developed by integrating glycidyl methacrylate with nanoparticles of bismuth oxide (Bi_2_O_3_) and tungsten oxide (WO_3_), were explored. The ability of the composites to attenuate gamma radiation was evaluated by measuring the emissions from Ba-133, Co-60, Cs-137, and Na-22. X-ray diffraction (XRD) spectra were obtained for both the pure polymer glycidyl methacrylate and the samples containing nanostructures of Bi_2_O_3_, Bi_2_O_3_/WO_3_, and WO_3_, and scanning electron microscopy (SEM) was used to analyze the samples. The incorporation of Bi_2_O_3_ and WO_3_ nanoparticles into the polymer glycidyl methacrylate matrix significantly enhanced the composites' ability to attenuate gamma radiation, as demonstrated by the increased linear and mass attenuation coefficients. The results showed good agreement between the experiment and the XCOM database. The composites exhibited significant efficiency in attenuating lower-energy gamma rays, which is particularly advantageous in the medical and nuclear industries.

## 1 Introduction

Electromagnetic radiation such as gamma rays, owing to its massless and uncharged nature, has high penetrating ability. Therefore, appropriate shielding materials must be used to protect individuals and the ecosystem from harmful radiation. Heavy metals, with their high atomic numbers and densities, are effective for gamma-ray shielding. However, their brittleness and poor mechanical properties limit their real-world applications, particularly when flexibility and long-term stability are required [[1], [2], [3], [4], [5], [6]]. To overcome these issues, researchers have recommended the use of reinforced polymer matrices to improve the flexibility, strength, and durability of heavy-metal compounds while maintaining their radiation-shielding effectiveness [7,8].

The use of nanoscale materials to fabricate reinforced polymers has improved their mechanical properties and radiation shielding efficiencies. Nanofillers, with their elevated surface-to-volume ratio, influence their molecular configurations. Incorporating nanofillers can enhance heat resistance, strength, stiffness, and reduce gas permeability. Furthermore, the addition of nanofillers can expand the surface area and increase the rate of interaction between gamma rays and matter, thereby improving radiation absorption [7,9,10].

Alabsy et al. (2020) [11] compared cadmium oxide and lead oxide as fillers in high-density polyethylene for gamma-ray shielding and found that cadmium oxide was more effective for lower-energy rays, while lead oxide was better for higher-energy rays, with nanosized fillers outperforming microsized ones. In 2021, Alabsy et al. [12] investigated the gamma-ray attenuation and mechanical properties of PVC composites with PbO and CuO nanoparticles, finding that the nanoparticles enhanced both attenuation and mechanical performance compared to bulk composites. Similarly, Badawi et al. (2020) [13] theoretically evaluated epoxy resin-metal chloride mixtures for gamma-ray shielding and demonstrated improved attenuation over both pure epoxy and lead. El-Khatib et al. (2021) [14] studied gamma-ray shielding in recycled rPVC composites reinforced with PbO and CuO particles, noting that nanosized PbO and CuO significantly enhanced radiation protection. Obeid et al. (2022) [15] examined HDPE composites filled with varying sizes and fractions of tungsten oxide (WO_3_), discovering that both the size and weight fraction of WO_3_ significantly affected gamma-ray shielding.

Several studies have focused on the use of reinforcing polymer materials for radiation shielding. Abunahel et al. (2018) [16] developed a nanocomposite material to shield against X-rays, utilizing a polymer matrix combined with Bi_2_O_3_ nanoparticles. This configuration increased the X-ray radiation absorption ability of the polymer due to the dispersion of nanostructured Bi_2_O_3_ in the PVA matrix. Higgins et al. (2019) [17] found that incorporating HfO_2_ and WO_3_ nanoparticles increased the mass attenuation coefficients (MAC) of an epoxy resin; at 122 keV, the MAC of both nanocomposites was five times higher than that of pure epoxy, and at 1112 keV and 1407 keV, the improvement was approximately 39.5–51.1 % and 15 %, respectively. These innovative nanocomposites offer improvements in mechanical, thermal, and chemical qualities while reducing reliance on lead-based materials. Abdalsalam et al. (2020) [8] prepared various concentrations of Bi_2_O_3_ nanopowders mixed with high-density polyethylene (HDPE) and exposed the samples to a focused gamma-ray beam at eight different energy levels ranging from 30.8 to 383.8 keV to determine the MAC. The addition of Bi2O3 nanopowder significantly improved the attenuation of gamma-ray shielding. Muthamma et al. (2021) [18] demonstrated a notable enhancement in the radiation-shielding efficiency of a Bi_2_O_3_ polymer composite by embedding bismuth oxide nanoparticles within a polymer matrix.

Moreover, several polymer matrices have been extensively examined for their effectiveness in radiation protection. These include composites such as poly (methyl methacrylate) (PMMA) reinforced with bismuth oxide (Bi_2_O_3_), HDPE composites loaded with molybdenum sulfide (MoS_2_), boron carbide (B4C), zinc oxide (ZnO), lead oxide (PbO), and cadmium oxide (CdO) [[19], [20], [21], [22], [23]].

In the present investigation, an approach toward synthesizing gamma-radiation-shielding composites was explored by employing a radical polymerization technique. The matrix contained glycidyl methacrylate (PGMA), Bi_2_O_3_, and WO_3_ nanopowders. The composites were tested for their shielding effectiveness against gamma energy levels, including Ba-133 (81, 303, and 356 keV), Co-60 (1173 and 1332 keV), Cs-137 (662 keV), and Na-22 (511 and 1274 keV). The aim was to fabricate a polymer containing Bi_2_O_3_ and WO_3_ nanoparticles to improve its radiation-shielding capabilities. This results in a cost-effective, easily constructed, and environmentally friendly gamma shield material. This type of shielding material offers full-range protection against gamma radiation, crucial in nuclear medicine, radiotherapy, and industrial applications.

## 2 Materials and methods

### 2.1 Synthesis of Bi_2_O_3_ NPs

A precipitation process was used to synthesize Bi_2_O_3_ NPs. 8 g of Bi(NO_3_)·5H_2_O was dissolved in 100 mL of nitric acid (HNO_3_). The resulting solution was then combined with urea in a molar ratio of 1:5 and heated in a water bath at 100 °C. A white precipitate was also observed. The solid substance was then decomposed at temperatures ranging from 400 to 700 °C. The powders exhibited an initial swelling behavior, filling the beaker and forming a foamy precursor. The foam consisted of uniformly distributed flakes with an average fine particle size of 80 nm.

### 2.2 Synthesis of WO_3_ NPs

The WO_3_ nanoparticles were produced using a hydrothermal method. 1.485 g of sodium chloride (NaCl) and 4 g of sodium tungstate dihydrate (Na_2_WO_4_·2H_2_O) were combined with 60 mL of distilled water. The mixture was then stirred for 30 min. During stirring, 16 mL of hydrochloric acid (HCl) was added incrementally. The solution was placed in an autoclave (100 mL Teflon-lined stainless steel) and heated at 140 °C for 24 h. The solution was then washed three times with ethanol and deionized water. Subsequently, the obtained materials were dried at 80 °C for 18 h, followed by a calcination process at 450 °C for 5 h.

### 2.3 Polymeric composites preparation

5 mL of glycidyl methacrylate (GMA) was polymerized by radical polymerization at 70 °C, using 2,2′-Azobis(2-methylpropionitrile) (AIBN) as the initiator, with a continuous flow of nitrogen gas. A high-viscosity solution formed after the reaction, confirming the formation of the polymer. An established quantity of Bi2O3-NPs (50 wt%) was introduced into the mixture, dispersed under stirring for 1 h, and subjected to sonication for 20 min to prevent the particles from clumping together. The final B-NPs/PGMA mixture was molded. The mold containing the sample was dried at 25 °C for 24 h. The W-NPs/PGMA and BW-NPs/PGMA polymer composites were prepared by repeating the procedures listed in Table 1. All the prepared samples had the same thickness of approximately 5 mm.

**Table 1:** Composition of PGMA, B-NPs/PGMA, W-NPs/PGMA, and BW-NPs/PGMA samples.

Sample code	Sample density (g/cm^3^)	Bi_2_O_3_-NPs (wt.%)	WO_3_-NPs (wt.%)	PGMA-matrix (wt.%)
PGMA	1.07	0	0	100
B-NPs/PGMA	1.91	50	0	50
W-NPs/PGMA	1.86	0	50	50
BW-NPs/PGMA	1.88	25	25	50

**Fig. 1:**
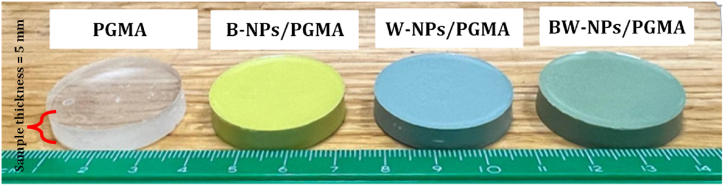
Fabricated polymer composite samples.

The densities of the prepared polymeric composites (Fig. 1) were theoretically calculated using equation (1) [24]:

(1) $$\rho_{\text{composite}}=\frac{1}{\sum \left(\frac{w_{i}}{\rho_{i}}\right)}$$

where:

$\rho_{\text{composite}}$ is the density of the composite material, $w_{i}$ is the weight fraction of the *i*th component, and $\rho_{i}$ is the density of the *i*th component.

### 2.4 Shielding parameters

LAC and MAC are basic properties of gamma rays that determine the absorbed or reduced radiation quantity as they traverse a substance, and are evaluated using equations (2), (3) [25,26]:

(2) $$LAC=\ln\left(\frac{I_{0}}{I}\right)/t$$

(3) $$MAC=\frac{LAC}{\rho }=\frac{\ln\left(\frac{I_{0}}{I}\right)}{t*\rho }$$

where “*I”* denotes the radiation intensity after passing through the layer “*t”* of the substance, whereas *I*_*0*_ denotes the initial intensity of the incoming radiation, and (ρ) is the material density.

The HVL is the matter thickness at which the photon intensity decreases by half after interacting with it. This is formally described by equation (4) [27]:

(4) $$HVL=\frac{\ln\left(2\right)}{LAC}$$

### 2.5 Gamma radiation attenuation measurement

**Fig. 2:**
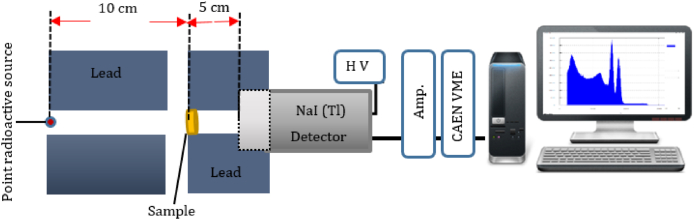
Scheme of the gamma-ray attenuation measurement system.

The gamma rays shielding ability of the polymeric composites was estimated by conducting experiments using a NaI(Tl) scintillation detector (BICRON: 2M2/2, Crystal Size: 2″ × 2″) and point radiation sources, namely Ba-133 (81, 303, and 356 keV) (9324 Bq), Co-60 (1173 and 1332 keV) (23643 Bq), Cs-137 (662 keV) (1.14 × 10⁵ Bq), and Na-22 (511 and 1274 keV) (5550 Bq). A CAEN controller (Model: V1718 VME) was used as the primary device. These instruments consisted of a CAEN analog-to-digital converter (ADC) (Model V785, 16/32), an amplifier (ORTEC, model: 485), and a gate generator (ORTEC, model: 416A). Fig. 2 shows the gamma-ray detection system setup used in this study. This establishes a fundamental measurement that can be used as a reference point for comparing the readings obtained using various types of shielding materials. Each measurement was taken for 20 min; to ensure accuracy, each measurement was performed three times, and the mean value was recorded.

The XCOM database, developed by the National Institute of Standards and Technology (NIST), was used to compare the theoretical mass attenuation coefficients with the experimental values. This database offers comprehensive photon cross sections and attenuation coefficients for elements, compounds, and mixtures over a broad energy range of 1–100 GeV [28].

### 2.6 Materials characterization

X-ray diffraction (XRD) was performed to ascertain the crystalline structure, phase composition, and crystallite size of the synthesized samples. The surface morphology and structural characteristics were examined using scanning electron microscopy (SEM).

## 3 Results and discussion

### 3.1 XRD analysis

**Fig. 3:**
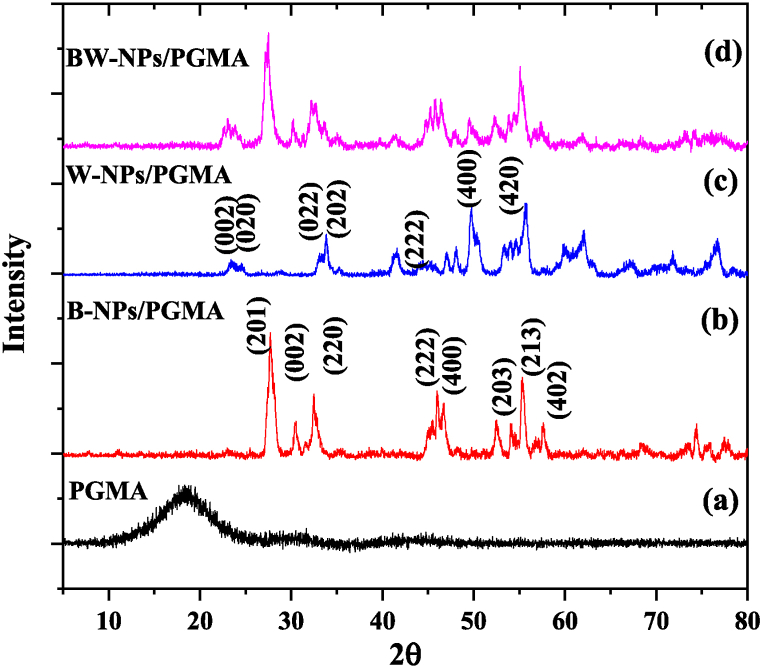
XRD pattern of the gamma ray attenuator samples (a) PGMA, (b) B-NPs/PGMA, (c) W-NPs/PGMA, (d) BW-NPs/PGMA.

Fig. 3(a) shows that the PGMA polymer was very broad, typical of any polymer or amorphous sample. Fig. 3(b) shows that the B-NPs/PGMA sample had peaks (corresponding to lattice points) at 27.6°(201), 30.48°(002), 32.39°(220), 47.09°(222), 52.94°(400), 55.51° (213), and 57.91° (402). The XRD pattern of B-NPs/PGMA was similar to that reported previously [29]. The XRD pattern of the W-NPs/PGMA sample contained several peaks. The broad peak around 22.94° corresponds to the (002) phase. Similarly, phases such as (020), (022), (202), (222), (400), and (420) [30] were observed. The XRD pattern of BW-NPs/PGMA shows peaks for both B-NPs and W-NPs, indicating the formation of a BW-NPs/PGMA composite. The relative intensity of the polymer was low for the composite sample, which could be due to the improved filling of the interstitial spaces, enhanced crystallinity, and sample homogeneity.

### 3.2 Morphology

**Fig. 4:**
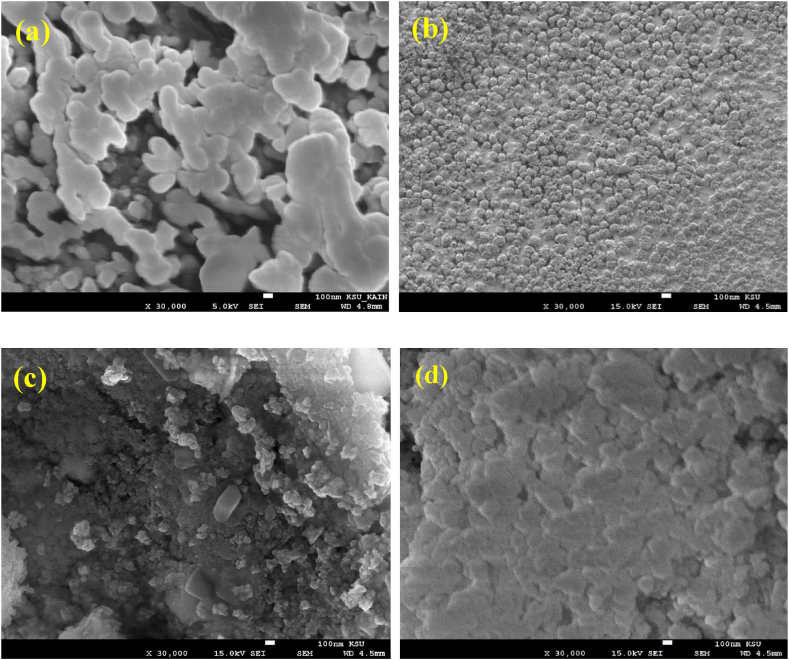
SEM images of (a) PGMA, (b) B-NPs/PGMA, (c) W-NPs/PGMA, (d) BW-NPs/PGMA.

Fig. 4 (a) shows SEM image of PGMA, which displayed round beads with an average size of 220 nm. The beads were nested together. However, Fig. 4 (b) showed B-NPs/PGMA nanoparticles with an average size of 80 nm, and most of the particles were less than 100 nm. SEM image in Fig. 4 (c) shows the structure of rhombus-shaped particles with an average size of 60 nm and a few large particles with lengths greater than 200 nm. The SEM images of BW-NPs/PGMA are depicted in Fig. 4(d), where the surface is very flat and aggregates of various shapes are found. The blend of B-NPs and W-NPs in an equal ratio to PGMA completely changed the morphology of the sample.

### 3.3 Radiation shielding properties

**Fig. 5:**
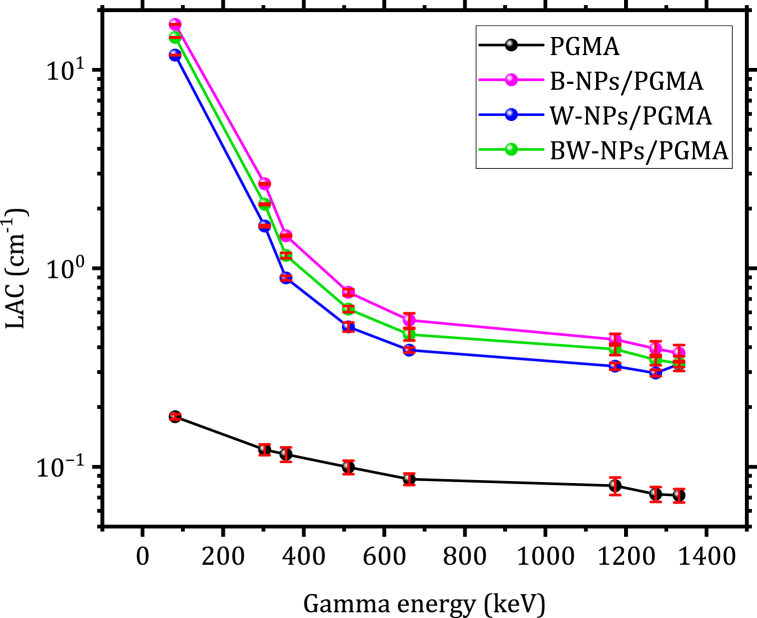
LAC vs. gamma energy at different prepared PGMA, B-NPs/PGMA, W-NPs/PGMA, and BW-NPs/PGMA filled composites.

The produced reinforced polymer materials exhibited a significant increase in LAC values within the lower energy range. This characteristic makes these composites appropriate for radiotherapy, where low-energy gamma rays and X-rays are dominant [31]. The reinforced polymer B-NPs/PGMA composite showed an effective increase in the LAC of gamma radiation at low energies (<200 keV) compared to the reinforced polymer composites W-NPs/PGMA and BW-NPs/PGMA with the same weight ratio (50 wt%). Moreover, as the gamma energy exceeded 800 keV, the differences between the LAC values of B-NPs/PGMA and other composites began to decrease until they were almost equal, continuing in this manner until the high energies of gamma radiation (>1100 keV), as shown in Fig. 5. This behavior can be explained by the interaction between gamma radiation and the nanocomposite materials, as well as the characteristics of the NPs [7,9]. The Compton scattering process becomes the dominant mechanism for gamma radiation attenuation at intermediate energies greater than 300–1000 keV. The possibility of Compton scattering is influenced by the electron density and the atomic number (Z-number) of the material [32].

Fig. 5. The LAC values decreased as the gamma-ray energy increased, attributed to the lower probability of high-energy photons interacting with matter. However, the decreasing LAC values can be divided into two phases: one at low energy and the other at high energy levels. The sharp decrease observed at lower energies is due to the photoelectric absorption of photons by the polymer composites. Owing to the dominance of the Compton scattering mechanism, the LAC decreased more gradually at higher energies [30,[33], [34], [35]]. The LAC also increased with the addition of Bi_2_O_3_, WO_3_, and Bi_2_O_3_/WO_3_ NPs at each energy, as the nanocomposites increased the polymer sample density [27].

**Fig. 6:**
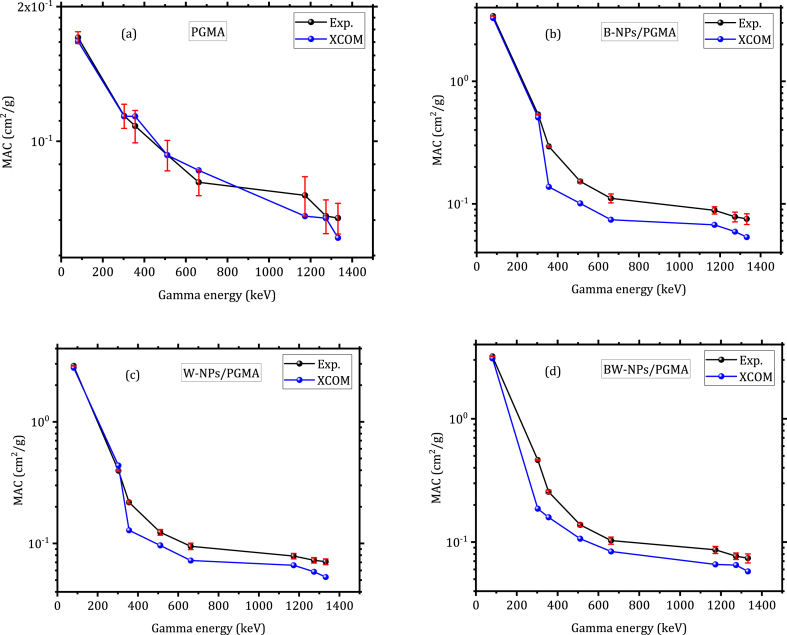
The experimental and XCOM MAC of the studied samples: (a) PGMA, (b) B-NPs/PGMA, (c) W-NPs/PGMA, (d) BW-NPs/PGMA.

The average experimental MAC values of the prepared polymeric samples are listed in Table 2. The findings demonstrated a significant decline in the MAC values of the composites containing different fillers at low gamma-energy regions, and only a small change was observed at higher gamma-ray energy levels. Furthermore, the filler loading of the nanocomposites had little effect on their shielding capabilities in high gamma energy regions; the nanocomposites exhibited only a small enhancement in attenuation efficiency as the gamma-ray energy increased, mainly attributed to the prevalence of Compton scattering and the heightened occurrence of pair production interactions [32] (Fig. 6; b, c, d).

**Table 2:** MAC values of PGMA, B-NPs/PGMA, W-NPs/PGMA, and BW-NPs/PGMA samples.

Gamma Energy (keV)									
Sample code		81	303	356	511	662	1173	1274	1332
MAC (cm^2^/g)	PGMA	0.167 ±0.0052	0.114 ±0.0071	0.108 ±0.0090	0.093 ±0.0073	0.081 ±0.0054	0.075 ±0.0076	0.068 ±0.0059	0.067 ±0.0053
	B-NPs/PGMA	3.402 ±0.0037	0.535 ±0.0045	0.293 ±0.0035	0.152 ±0.0056	0.110 ±0.0092	0.088 ±0.0059	0.079 ±0.0071	0.075 ±0.0075
	W-NPS/PGMA	2.882 ±0.0096	0.396 ±0.0029	0.217 ±0.0061	0.123 ±0.0066	0.094 ±0.0028	0.078 ±0.0031	0.072 ±0.0027	0.080 ±0.0028
	BW-NPs/PGMA	3.201 ±0.0073	0.462 ±0.0048	0.255 ±0.0082	0.137 ±0.0049	0.102 ±0.0069	0.086 ±0.0057	0.076 ±0.0046	0.073 ±0.0062

A comparison of the empirical and theoretical MAC values from the XCOM database is shown in Fig. 6(a)–(d). The findings from the experimental and theoretical approaches exhibited high agreement with little difference across all energy levels. It is evident from Fig. 6 and previous studies [31,33,36] that the prepared polymeric nanocomposites showed lower MAC values than the lead material shield at lower gamma energy levels due to the dominant reaction mechanism of the photoelectric effect on the Z value of the reactant matter. As the gamma energy increased, the attenuation dependence on Z declined, therefore, the MAC of the nanocomposites became more comparable to that of the lead shield [34,35,37,38]. The MAC shielding performance values of the nanocomposites fabricated in this study were compared with those described in the literature at an energy of 662 keV (Table 3). This energy level was selected for comparative analysis because it has been extensively investigated in previous research.

**Table 3:** Comparative shielding analysis of polymer composite MAC between this current study and data from literature at 662 keV gamma rays.

study	Material	MAC (cm^2^/g) at 662 keV
Current study	PGMA	0.081
	B-NPs/PGMA	0.110
	W-NPs/PGMA	0.095
	BW-NPs/PGMA	0.102
(Mahmoud et al., 2018a, b) [21]	HDPE	0.099
	HDPE-PbO 10 % bulk PbO	0.100
	HDPE-PbO 50 % bulk PbO	0.133
	HDPE-PbO 10 % bulk PbO nanoparticles	0.110
	HDPE-PbO 50 % bulk PbO nanoparticles	0.143
(M. Higgins et al., 2019) [17]	Epoxy	0.029
	HfO_2_-epoxy	0.044
	WO_3_-epoxy	0.040
(Alsayed et al., 2019) [23]	HDPE	0.070
	HDPE 10 %/bulk ZnO	0.068
	HDPE 20 %/bulk ZnO	0.067
	HDPE 30 %/bulk ZnO	0.065
	HDPE 40 %/bulk ZnO	0.065
	HDPE 10 %/bulk ZnO nanoparticles	0.072
	HDPE 20 %/bulk ZnO nanoparticles	0.077
	HDPE 30 %/bulk ZnO nanoparticles	0.078
	HDPE 40 %/bulk ZnO nanoparticles	0.077
(Muthamma et al., 2021) [18]	Nano Bi_2_O_3_ filled epoxy/nB2	0.085
	Nano Bi_2_O_3_ filled epoxy/nB4	0.085
	Nano Bi_2_O_3_ filled epoxy/nB6	0.087
	Nano Bi_2_O_3_ filled epoxy/nB8	0.088
	Nano Bi_2_O_3_ filled epoxy/nB10	0.090
	Nano Bi_2_O_3_ filled epoxy/nB12	0.094
	Nano Bi_2_O_3_ filled epoxy/nB14	0.094

**Fig. 7:**
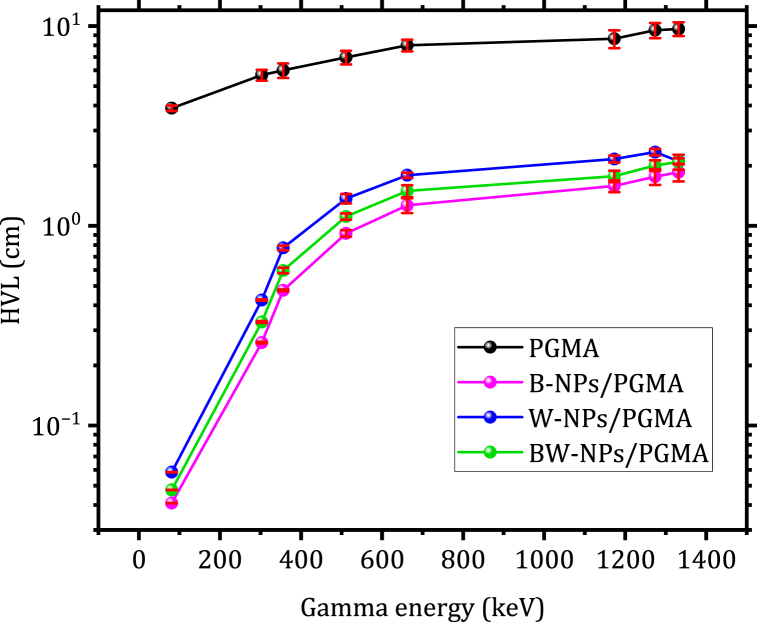
HVL as a function of gamma energy for the samples: PGMA, B-NPs/PGMA, W-NPs/PGMA, and BW-NPs/PGMA.

Fig. 7 illustrates how the HVL values vary with gamma energy across various filler loadings of Bi_2_O_3_, WO_3_, and Bi_2_O_3_/WO_3_ NPs in the polymer matrix. The HVL is a crucial parameter in the evaluation of fabricated shielding. These results suggest that a greater thickness is necessary to effectively attenuate high-energy photons. Additionally, the HVL value at 662 keV for pure PGMA was reduced by 576 %, 411 %, and 492 % after being filled with B-NPs, W-NPs, and BW-NPs, respectively. Consequently, the observed HVL values for B-NPs/PGMA confirm its heightened attenuation characteristics compared to other compositions.

## 4 Conclusion

This study investigated the shielding properties of novel polymer composites developed using glycidyl methacrylate with bismuth and tungsten oxide nanoparticles. The results demonstrated that the reinforced polymer composite, namely B-NPs/PGMA, exhibited a notable enhancement in the linear and mass attenuation coefficients of gamma radiation at energies below 200 keV. The results also showed a consistent increase in the half-value layer (HVL) of the B-NPs/PGMA samples as gamma ray energy increased. More precisely, the half-value layer for the B-NPs/PGMA sample increased from 0.0412 cm at 81 keV to 1.265 cm at 662 keV, and rose to 1.864 cm at 1332 keV. Conversely, the HVL at 662 keV of pure polymer glycidyl methacrylate decreased by 576 %, 411 %, and 492 % when strengthened with B-NPs, W-NPs, and BW-NPs, respectively. The aforementioned results promote the use of these materials for many shielding applications in the medical (radiotherapy and nuclear medicine) and environmental fields. This research contributes to the advancement of nanotechnology-based shielding materials with diverse uses, such as in medical radiation therapy and nuclear power.

## Data availability statement

Data available on request.

## CRediT authorship contribution statement

**Hamoud Kassim:** Writing – original draft, Methodology. **Saad Aldawood:** Writing – review & editing, Supervision, Resources, Funding acquisition. **Saradh Prasad:** Writing – original draft. **Nassar N. Asemi:** Methodology, Data curation. **Aziz A. Aziz:** Methodology. **Mohamad S. AlSalhi:** Investigation.

## Declaration of competing interest

The authors declare that they have no known competing financial interests or personal relationships that could have appeared to influence the work reported in this paper.
